# Cross Sectional Analysis of Impact of Seasonal Changes on Excimer Laser Ablation Performance on Polymethyl Methacrylate (PMMA)

**DOI:** 10.3390/vision7030050

**Published:** 2023-07-14

**Authors:** Shwetabh Verma, Juergen Hesser, Samuel Arba Mosquera

**Affiliations:** 1 Biomedical Engineering Office, Research and Development, SCHWIND Eye-Tech-Solutions, 63801 Kleinostheim, Germany; shwetabh.verma@eye-tech.net; 2 Interdisciplinary Center for Scientific Computing (IWR), Heidelberg University, 69117 Heidelberg, Germany; juergen.hesser@medma.uni-heidelberg.de; 3 Mannheim Institute for Intelligent Systems in Medicine (MIISM), Heidelberg University, 69117 Heidelberg, Germany; 4 Central Institute for Computer Engineering (ZITI), Heidelberg University, 69117 Heidelberg, Germany; 5 CZS Heidelberg Center for Model-Based AI, Heidelberg University, 69117 Heidelberg, Germany

**Keywords:** impact of seasonal variations, humidity, temperature, excimer laser ablation, PMMA, cornea, PET, refractive surgery

## Abstract

Seasonal changes and varying degree of corneal hydration has been linked to excimer laser corneal ablation rates. The use of PMMA as a calibration material in refractive lasers is well established. However, PMMA ablation may be equally affected by seasonal variations in temperature and humidity, in turn affecting the calibration process. The aim of this work is to analyze the effect of seasonal changes in PMMA performance using SCHWIND AMARIS laser system. PET and PMMA ablations conducted in climate-controlled environment with 826 consecutive AMARIS systems manufactured over 6 years were retrospectively analyzed. Lasers were stratified depending on seasons and months of the year. Metrics like single laser pulse fluence, nominal number of laser pulses, mean performance, standard deviation, and technical performance of system were compared to global average values. Cyclic winter–summer variation was confirmed with seasons winter and summer showing statistically significant variations with respect to global values. Metric technical performance showed deeper PMMA ablation performance in summertime, with maximum seasonal deviation of 6%. Results were consistently confirmed in seasonal as well as monthly analyses. These findings could help minimize variance among laser systems by implementing compensation factors depending on seasons such that laser systems installed worldwide follow the same trend line of variation.

## 1. Introduction

Laser based refractive surgery involves application of laser pulses on the corneal tissue, performed in a temperature and humidity-controlled environment. However, despite strong climatic controls, the varying degree of corneal hydration is linked to excimer laser corneal ablation rates [1,2]. Several groups have tested the impact of hydration and room temperature in human eyes undergoing refractive surgery in the past, reporting different influences on post-ablation outcomes [3,4,5,6,7,8,9]. Luger et al. [10] reported seasonal differences in residual refraction 1-year after corneal refractive surgery among a large-scale population. Using univariate and multivariate analysis, it has been found that LASIK enhancement rates strongly correlated with procedure room humidity, 2-week preparative mean outdoor humidity, outdoor temperature, and age; suggesting that these factors should be taken into account while planning the LASIK procedure [11]. In another study, a modified LASIK procedure was performed on the corneal surface that was kept relatively dry by blotting of the stromal surface between sets of laser pulses [12]. It was reported that for less hydrated corneas, ablation effects were greater than for corneas not blotted during the LASIK procedure, but these patients appeared to undergo greater myopic regression. Contrary results have also been reported with other laser platforms where no significant difference could be identified in patients grouped according to season at time of treatment [13].

It is imperative to calibrate and maintain refractive laser systems to repeatedly deliver the same standards of performance over their entire life cycle [14,15]. Laser systems often utilize plastic material ablations for calibration [14] and optimization of system parameters [16]. The use of Polymethylmethacrylate (PMMA) as a calibration material has been well analyzed in the past, and used to develop comprehensive models, considering applied correction, as well as laser beam characteristics and ablative spot properties [17,18,19,20,21]. However, PMMA ablation may also be affected by the variations in temperature and humidity [22,23].

Our aim with this large-scale retrospective cross-sectional study is to quantitatively analyze the effect of seasonal variations of temperature and humidity despite the controlled room environment conditions, on the performance of excimer laser ablation of PMMA using a large series of SCHWIND AMARIS laser systems (SCHWIND eye-tech-solutions, Germany).

Since PMMA is commonly used as a calibration material in refractive laser systems, this analysis could shine light on the influence of seasonal changes on the clinical outcomes of refractive surgery using this laser platform and in general.

## 2. Materials and Methods

The performance of an AMARIS laser system is evaluated through a series of Polyethylene Terephthalate (PET) and PMMA ablations (PMMA test) as a final check for the system under production, in order to qualify the system as ready for clinical use. The PET ablations are used to determine the number of laser pulses required to ablate a PET foil of specified thickness and ablation properties, whereas PMMA ablations are used to determine the ablation performance by comparing the achieved ablation to the planned ablation. These series of ablations are repeated throughout the lifetime of a laser system with a defined frequency to calibrate the system to a nominal performance level. Generally, a higher single laser pulse fluence and a greater number of laser pulses lead to deeper ablations in PMMA and ideally a similar behaviour during ablation of the cornea tissue. Different metrics are useful to identify trends and variability in Laser system performance. For the SCHWIND AMARIS system, the relevant parameters are described in Table 1 with their typical values and potential impacts on system performance.

In this study, 826 consecutive AMARIS systems (including models 500E, 750S, 1050RS) manufactured from February 2012 to February 2018 (6 years) were retrospectively reviewed. The distribution of the analyzed systems according to seasons and months is presented in Table 2.

For every system, PET and PMMA ablations performed as a final check in production were considered, to quantitatively analyze the effect of seasonal changes on the performance of excimer laser ablation of PET and PMMA. Since all the system adjustments are already conducted to determine the nominal fluence and pulse energy parameters prior to performing the final system check, it is assumed for the sake of this analysis that only the influence of seasonal change affected the ablation performance. All the ablations were conducted in climate-controlled environment at the SCHWIND production facility in Kleinostheim, Germany. Although the room conditions were set to a constant value, a small range of deviation in the set temperature and relative humidity could not be avoided. Since this deviation (21–24 °C temperature and 30–50% Relative humidity) was very small compared to the variations in temperature and relative humidity due to the seasonal changes, the isolated influence of the seasonal changes could still be evaluated.

Seasonal outcomes were evaluated in terms of PMMA and PET Performance stratified for every month in a year, as well as stratified for each season in a year. The seasons were defined with respect to the calendar months as winter (January to March), spring (April to June), summer (July to September), and autumn (October to December).

Statistical Analysis

- Since the standard PMMA test comprises of 12 different ablations [15], for each laser system the mean performance and standard deviation in all the ablations was calculated.
- There are two energy levels used in the AMARIS laser system (High Fluence value of 1.0 mJ (~450 mJ/cm^2^) and Low Fluence value of 0.7 mJ (~300 mJ/cm^2^)). For each laser system, nominal number of laser pulses (determined with PET ablations) and nominal single laser pulse fluence (determined with PMMA ablations) were recorded for both the energy settings.
- Based on the distribution of the two energy settings in the AMARIS laser system, and the measured nominal single pulse fluence and nominal number of laser pulses, the overall system performance was calculated for each laser system and termed as the ‘technical’ performance of the system.
- The parameters analyzed in the following steps were: nominal single laser pulse fluence (high fluence and low fluence), nominal number of laser pulses (high fluence and low fluence), mean performance (in 12 PMMA ablations per system), standard deviation (in 12 PMMA ablations per system), and technical performance of the system (comprising distribution of the two energy levels). For each of these parameters, the global average was calculated as the average value obtained for all the laser systems under analysis.
- The lasers were stratified depending on the season of the year and depending on the months of the year.
- Student’s T-test was performed to statistically compare the stratified values of the parameters based on the season/month of the year, with the global average values of the parameters. The level of statistical significance was set to *p* ≤ 0.05. The normality of the data was verified through ‘back of the envelope test’.
- Percentage deviation in stratified values of each parameter was calculated with respect to the global average values. Based on the deviation, underperformance (meaning relatively shallower ablation, less-than-planned correction) or overperformance (meaning relatively deeper ablation, more-than-planned correction) in terms of ablation was assessed.

## 3. Results

### 3.1. Seasonal Influence

**Figure 1:**
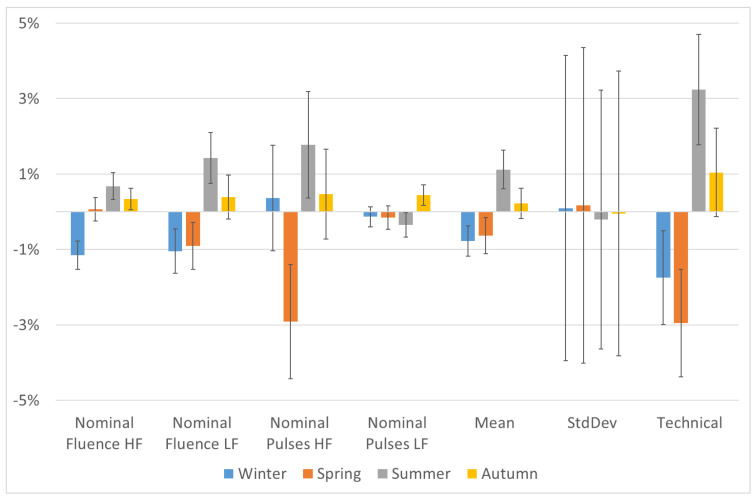
The percentage deviation in the average values of all the parameters in each season with respect to their global averages is presented individually. Here, ‘nominal fluence’ represents single laser pulse fluence, ‘HF’ is high fluence, ‘LF’ is low fluence, ‘StdDev’ is standard deviation, and the different seasons were defined with respect to the calendar months as, winter (January to March), spring (April to June), summer (July to September), and autumn (October to December).

**Figure 2:**
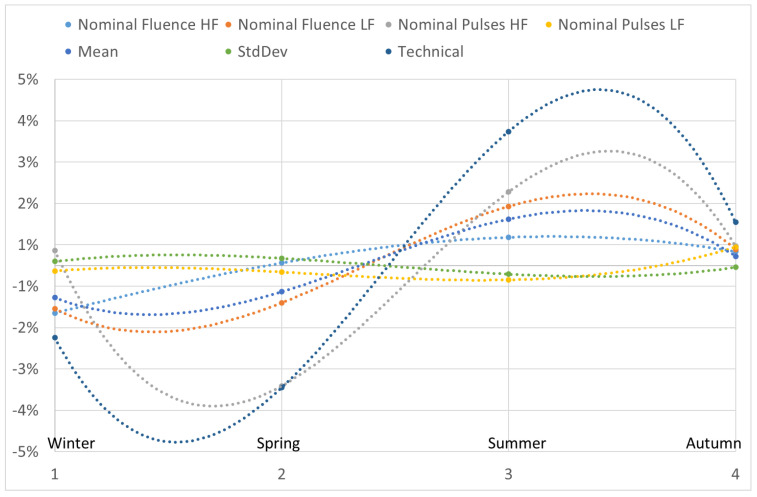
The percentage deviation in the average values of all the parameters in each season with respect to their global averages is presented as a group. Here, ‘nominal fluence’ represents single laser pulse fluence, ‘HF’ is high fluence, ‘LF’ is low fluence, ‘StdDev’ is standard deviation, and the different seasons were defined with respect to the calendar months as winter (January to March), spring (April to June), summer (July to September), and autumn (October to December).

The percentage deviation in the average values of all the parameters in each season with respect to their global averages is presented individually in Figure 1 and as a group in Figure 2. All the parameters showed a strong cyclic trend with the changing season, with the most predominant change observed for winter and summer season. A third order polynomial fit shows the variability and trend in the analyzed metrics. The percentage deviation in the parameter ‘standard deviation’ among the 12 PMMA ablations was particularly higher compared to the other parameters; however, this was due to the fact that percentage changes were examined in numbers of originally very low magnitude.

Although a cyclic trend can be observed for all the parameters among the various seasons, not all the observed changes reached statistical significance. The seasons winter and summer showed statistical significance with respect to the global average values for all the tested parameters except the nominal number of laser pulses for high and low fluence setting. The trends showing statistical significance and the range or maximum deviation for each parameter is presented in Table 3. The metric technical performance of the analyzed systems showed a stronger PMMA ablation performance (meaning deeper PMMA ablations) in Summer time compared to a weaker performance in the wintertime, with the maximum seasonal deviation of 6%.

### 3.2. Seasonal Influence-Stratification on Months of a Year

**Figure 3:**
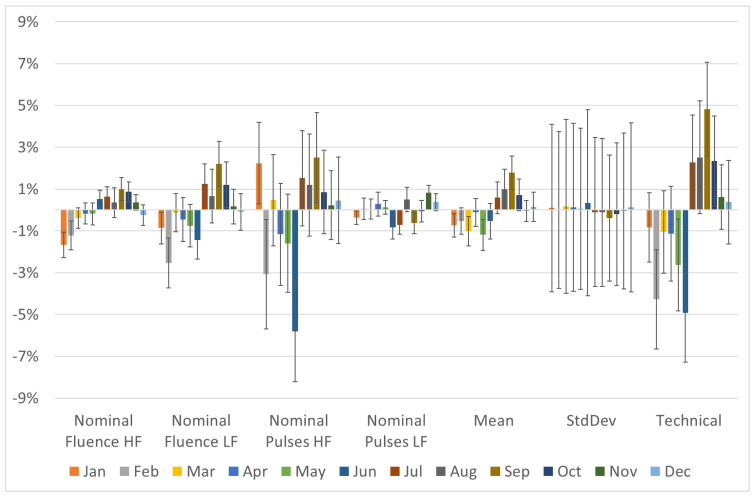
The percentage deviation in the average values of all the parameters in each month of the year with respect to their global averages is presented individually. Here, ‘Nominal fluence’ represents Single laser pulse fluence, ‘HF’ is high fluence, ‘LF’ is low fluence, ‘StdDev’ is standard deviation.

**Figure 4:**
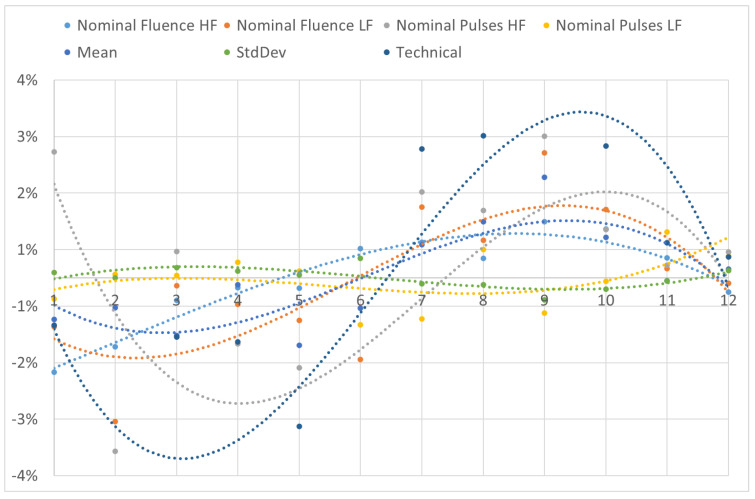
The percentage deviation in the average values of all the parameters in each month of the year with respect to their global averages is presented as a group. Here, ‘Nominal fluence’ represents Single laser pulse fluence, ‘HF’ is high fluence, ‘LF’ is low fluence, ‘StdDev’ is standard deviation.

The percentage deviation in the average values of all the parameters in each month of the year with respect to their global averages is presented individually in Figure 3 and as a group in Figure 4. Even upon stratifying the lasers depending on the month of the year, a similar cyclic trend as observed among the four seasons could be reproduced consistently, confirming the findings of the analysis. A third order polynomial fit shows the variability and trend in the analyzed metrics. Similar to the seasonal deviations, the percentage of monthly deviation in the parameter ‘standard deviation’ among the 12 PMMA ablations with respect to the global values was particularly high compared to the other parameters due to the very low magnitude.

The trend showing statistical significance among the different months of a year, and the range or maximum deviation for each parameter is presented in Table 4.

## 4. Discussion

Attempts have been made in the past to analyze the variation in PMMA ablations. Dantas et al. [3] evaluated excimer laser fluence after experimentally induced changes in room temperature and relative air humidity and concluded that in a setting with controlled temperature and relative air humidity, subtle changes in environmental factors do not appear to influence laser fluence and efficacy but acknowledge that the variations seen in PMMA test ablations may not translate completely into clinical changes. Regarding tissue characteristics and specificity, stromal tissue may be more sensitive to environmental changes than PMMA because of the differences in ablation thresholds and the effects of dehydration.

In this cross-sectional study, the seasonal PMMA performance of over 826 laser systems was analyzed over 6 years. The comparison of stratified average values (seasonal or monthly) of the parameters with the global average values of the parameters for the entire dataset of lasers was preferred as a more restrictive and robust statistical analysis approach. Our results demonstrated a cyclic winter–summer variation in both PET and PMMA ablation performance, and hence the technical performance of the system.

In our methods, some of the performance parameters (e.g., nominal pulses HF and LF) are integers and correspond to discrete variables. In a strict sense, these may not comply with the theoretical assumptions of the t-test. However, since the number of pulses analyzed are large values (between ~10,000 and ~12,000 pulses) compared to the unitary step (single pulse), the central limit theorem validates the use of t-test for with the analyzed data and metrics.

We statistically compared the seasonal metrics with the global metrics using a t-test. Although the sizes of the compared data are not equal (826 systems globally vs. as little as 180 systems in the spring season) to follow the theoretical assumption of the t-test, the square root of the analyzed sample sizes (effect size as 1/sqrt(n)) is twice apart. In addition, the global data set also includes the seasonal group being compared, squeezing the differences even further. Alternatively, a comparison using a 12 × 12 matrix could be too confounding to be intuitively interpreted, while other statistical tests like ANOVA could be too global to draw conclusions.

Luger et al. [10] analyzed the seasonal clinical ablation performance in a longitudinal study of two SCHWIND AMARIS laser systems used by 12 surgeons over 2 years (5740 treatments). Their results showed that treatments performed in April, June, August, September, and October showed relative under-corrections of the spherical equivalent (SE) (−0.09D), whereas treatments performed in January, February, and March showed relative overcorrections of the SE (+0.13D). Similarly, treatments performed in spring and summer showed relative under-corrections of the SE (−0.04D), whereas treatments performed in winter showed relative overcorrections of the SE (+0.10D). Compared to these clinical findings showing a weaker performance in summertime (under-correction) and stronger performance in wintertime (overcorrection), our analysis showed an opposite trend in the performance on PMMA. In our results, the range or maximum deviation in PMMA performance from winter to summer was ~+6%; comparatively, Luger et al. reported that the range in clinical ablation performance from winter to summer was ~−5%. Both these deviations may have the same explanation, since calibrating a system that is overperforming on a test material (with ~+6% over performance on PMMA in this case) in winter season and bringing this system to the baseline (down to zero) in winter, may result in a corresponding underperformance of the same magnitude on cornea during the summer season.

A comparison of PMMA and porcine corneal tissue in terms of the influence of temperature and relative humidity, was presented in a study testing the impact of a wide range of temperature (~18 °C to ~30 °C) and relative humidity (~25% to ~80%) on laser ablation outcomes using nine climate test settings in a climate chamber [24]. The results of this study were based on tests conducted with one laser system used over a series of days of testing, and under a range of climate conditions. Thus, the inter-system variability could not be analyzed. This study also reported an opposite trend between the performance on PMMA and porcine cornea, where moist (80% Relative humidity) and cold (18 °C) climate conditions showed higher ablation performance in PMMA, while dry (20% relative humidity) and hot (30 °C) conditions favored an improved ablation performance on porcine corneal tissue. In this study, the range or maximum deviation in PMMA performance was reported ~5%, while the range in porcine corneal tissue ablation performance was ~12%. The comparison of our findings with the clinical results published by Luger et al. [10] using the same laser platform also confirmed the opposite trends followed by PMMA and corneal tissue ablation under different seasonal conditions. However, the ranges or maximum deviation in performance observed in our series are wider than the results reported from the climate chamber tests, especially considering the controlled room conditions with subtle seasonal changes in our settings compared to the forced extreme conditions in the climate chamber tests (temperature ~18 °C to ~30 °C and relative humidity ~25% to ~80%). This finding of an opposite trend in performance of PMMA and tissue is counterintuitive, as principally, the behavior on calibration material is expected to follow the same trend as the ablation of the target tissue. These opposite trends may amplify each other, since calibrating in summer season could make the laser underperform on PMMA, which after potentially being adjusted to 100%, would result in over-performance on the cornea.

In our results, the correspondence between nominal laser pulses (determined on PET) and the nominal single laser pulse fluence (determined on PMMA) may indicate that the underlying effect is not arising from the ablation material but from the laser system itself. Furthermore, our results were consistently confirmed in the seasonal as well as monthly analyses showing a very comparable trend. Although all the ablations were performed with laser systems under stable room conditions and only minor seasonal differences in relative humidity, differences in performance could be observed. Since the recorded room temperature and relative humidity remained almost constant throughout the entire duration of the test (21–24 °C (21–22 °C in winter and 23–24 °C in summer) and 30–50% Relative humidity (30–40% in winter and 40–50% in summer)), this may indicate that not only does the room temperature and relative humidity play a role in influencing the laser system performance, but other factors may also be involved like the air circulation which may be affected by seasonal variations (blowing hot air in wintertime vs. cold air and dehumidifying in summertime).

As per the design of SCHWIND AMARIS, the output of PET ablations calibrates the PMMA ablations in the short term (interval of 2 h). Therefore, the internal compensation of SCHWIND AMARIS (preset energy, pulses) could be a factor for reducing the influence of temperature and relative humidity on PMMA ablation. In any modern commercially available refractive laser system, calibration cycles are repeated at different frequencies depending on the frequency of the system feedback [15]. In one form or another, since all laser systems follow the same principle of short-term calibration, the results of our tests on PMMA and porcine cornea, and their interrelationship would remain valid.

The difference in altitude affecting the performance of laser systems, could not be analyzed (single facility). Additionally, it would have been interesting to correlate our findings with the actual room conditions (temperature, humidity, air flow, etc.); however, due to the retrospective nature of this study, this data could not be extracted for further analysis. This can be regarded as a potential limitation of our methods. A cross sectional analysis is generally limited in analyzing behavior over a period, furthermore, to determine a cause-and-effect relationship. It is only effective when it represents the entire population and has a large enough sample size to provide accuracy. We analyzed the ablation performance with only SCHWIND AMARIS laser platform, with all the three commercially available models combined in a single cohort. This can be limiting in providing conclusive evidence for the influence of seasonal changes on excimer lasers in general. Nevertheless, due to the longitudinal nature of the study, lasting six years and analyzing a large number of AMARIS systems, a scientific conclusion can be drawn with reasonable confidence for the AMARIS laser platform.

In summary, this large-scale retrospective cross-sectional study demonstrated a cyclic winter–summer variation in PMMA ablation using the SCHWIND AMARIS lasers. The results were consistently confirmed in seasonal as well as monthly analyses. A third order polynomial, being the Taylor expansion of the sine function, was used to fit the data and depict the variability and cyclic trend. The cycling variation is reinforced considering that the month wise analysis still preserves the phase seen in the seasonal analysis (Figure 2 and Figure 4). For example, the peaks seen in March/April and September/October months correspond to the winter/spring and summer/autumn transitions. Since the results reported here and the existing evidence suggests a cyclic variation due to seasonal affects [24], a compensation factor depending on the season of the year can be implemented in the systems coming out of production, to help minimize the variance among the laser systems and ensure that all laser systems installed worldwide follow the same trend line of variation. Due to the prevalence of PMMA as a calibration material for many commercially available excimer laser platforms, the findings could be also interpreted and applied to other systems as well. Most clinical laser systems and surgeons nowadays use refined nomogram to help with under-correction and overcorrection based on many factors. Our results suggest that the seasonal variations could also be a factor in various systems behaving differently depending on their time of production. With the proposed seasonal compensation factor, a better harmonization in the nomogram developed and implemented at various clinical sites could be also expected. Nevertheless, the underlying mechanism for the variations seen especially in climate-controlled settings with subtle seasonal variations, remains unexplained and warrants further exploration. Furthermore, the relationship between calibration materials like PMMA and corneal tissue shall be analyzed cautiously to optimize the calibration routine.

## Figures and Tables

**Table 1:** Metrics used to evaluate the performance of AMARIS laser systems, with their description, Typical Values and their impact on performance.

Metric	Description	Typical Value (Units)	Impact
Nominal fluence HF	Calibrated spot depth of high energy pulse (1.0 mJ)	710 nm	Lower values result in more pulses for a given treatment → Over correction
Nominal fluence LF	Calibrated spot depth of low energy pulse (0.7 mJ)	570 nm	Lower values result in more pulses for a given treatment → Over correction
Nominal pulses HF	Calibrated number of high energy pulses required to go through the test material (PET)	12,300 pulses	Lower values result in more pulses for a given treatment → Over correction
Nominal pulses LF	Calibrated number of low energy pulses required to go through the test material (PET)	10,330 pulses	Lower values result in more pulses for a given treatment → Over correction
Mean Performance	Ratio between achieved and attempted refractive correction on test material (PMMA)	100%	Lower values → Under correction
Standard Deviation	Standard deviation of the achieved vs. attempted refractive correction on test material (PMMA)	<4.0%	Lower values → Less variability
Technical Performance	Ratio between actual and theoretical performance on test material (PMMA)	100%	Lower values → Under Performance

**Table 2:** Distribution of the number of analyzed AMARIS systems according to months and seasons of the year.

Months	Number of AMARIS Systems Analyzed	Seasons	Number of AMARIS Systems Analyzed
January	85	Winter	196
February	48		
March	63		
April	56	Spring	180
May	62		
June	62		
July	68	Summer	184
August	51		
September	65		
October	76	Winter	266
November	118		
December	72		

**Table 3:** The trends showing statistical significance and the range or maximum deviation for each parameter, analyzed in lasers stratified depending on the season of the year.

Value	Single Laser Pulse Fluence (HF)	Single Laser Pulse Fluence (LF)	Nominal Pulses (HF)	Nominal Pulses (LF)	Mean	Standard Deviation	Technical Performance
Season where the value was Lower than Global Average (*p* < 0.05)	Winter	Winter	Spring	Stable	Winter	Summer	Winter
Season where the value was higher than Global Average (*p* < 0.05)	Summer	Summer	Summer	Stable	Summer	Winter	Summer
Range or maximum deviation	2%	2%	5%	1%	2%	0%	6%

**Table 4:** The trend showing statistical significance and the range or maximum deviation for each parameter, analyzed in lasers stratified depending on months of the year.

Value	Single Laser Pulse Fluence (HF)	Single Laser Pulse Fluence (LF)	Nominal Pulses (HF)	Nominal Pulses (LF)	Mean	Standard Deviation	Technical Performance
Months where the value was Lower than global average (*p* < 0.05)	January–February	January–June	February–June	Stable	January–June	July–October	January–June
Months where the value was higher than global average (*p* < 0.05)	June–October	July–October	July–October	Stable	July–October	January–June	July–October
Range or maximum deviation	3%	5%	9%	1%	3%	1%	10%

## Data Availability

The data presented in this study are available on request from the corresponding authors. The data are not publicly available due to confidentiality restrictions.
